# Bismuth sulfoiodide (BiSI) nanorods: synthesis, characterization, and photodetector application

**DOI:** 10.1038/s41598-023-35899-7

**Published:** 2023-05-31

**Authors:** Krystian Mistewicz, Tushar Kanti Das, Bartłomiej Nowacki, Albert Smalcerz, Hoe Joon Kim, Sugato Hajra, Marcin Godzierz, Olha Masiuchok

**Affiliations:** 1 Institute of Physics - Center for Science and Education, Silesian University of Technology, Krasińskiego 8, 40-019 Katowice, Poland; 2 Department of Industrial Informatics, Faculty of Materials Science, Joint Doctorate School, Silesian University of Technology, Krasinskiego 8, 40-019 Katowice, Poland; 3 Department of Industrial Informatics, Faculty of Materials Science, Silesian University of Technology, Krasinskiego 8, 40-019 Katowice, Poland; 4 Department of Robotics and Mechatronics Engineering, Daegu Gyeongbuk Institute of Science and Technology, Daegu, 42988 Republic of Korea; 5 Centre of Polymer and Carbon Materials, Polish Academy of Sciences, M. Curie-Skłodowskiej 34, 41-819 Zabrze, Poland; 6 International Polish-Ukrainian Research Laboratory Formation and Characterization of Advanced Polymers and Polymer Composites (ADPOLCOM), Kyiv, Ukraine; 7 E.O. Paton Electric Welding Institute, National Academy of Sciences of Ukraine, 11 Kazymyr Malevych Str, Kyiv, 03680 Ukraine

**Keywords:** Chemical engineering, Electrical and electronic engineering, Materials for devices, Nanoscale materials, Electronic properties and materials, Synthesis and processing, Nanosensors

## Abstract

The nanorods of bismuth sulfoiodide (BiSI) were synthesized at relatively low temperature (393 K) through a wet chemical method. The crystalline one-dimensional (1D) structure of the BiSI nanorods was confirmed using high resolution transmission microscopy (HRTEM). The morphology and chemical composition of the material were examined by applying scanning electron microscopy (SEM) and energy-dispersive X-ray spectroscopy (EDS), respectively. The average diameter of 126(3) nm and length of 1.9(1) µm of the BiSI nanorods were determined. X-ray diffraction (XRD) revealed that prepared material consists of a major orthorhombic BiSI phase (87%) and a minor amount of hexagonal Bi_13_S_18_I_2_ phase (13%) with no presence of other residual phases. The direct energy band gap of 1.67(1)  eV was determined for BiSI film using diffuse reflectance spectroscopy (DRS). Two types of photodetectors were constructed from BiSI nanorods. The first one was traditional photoconductive device based on BiSI film on stiff glass substrate equipped with Au electrodes. An influence of light intensity on photocurrent response to monochromatic light (λ = 488 nm) illumination was studied at a constant bias voltage. The novel flexible photo-chargeable device was the second type of prepared photodetectors. It consisted of BiSI film and gel electrolyte layer sandwiched between polyethylene terephthalate (PET) substrates coated with indium tin oxide (ITO) electrodes. The flexible self-powered BiSI photodetector exhibited open-circuit photovoltage of 68 mV and short-circuit photocurrent density of 0.11 nA/cm^2^ under light illumination with intensity of 0.127 W/cm^2^. These results confirmed high potential of BiSI nanorods for use in self-powered photodetectors and photo-chargeable capacitors.

## Introduction

Bismuth sulfoiodide (BiSI) is a ternary semiconductor that belongs to the chalcohalide family of inorganic materials^1,2^. The crystal structure of the BiSI is described by the orthorhombic *Pnam* space group^3,4^. This material grows into the needle-shaped bulk crystals^5–7^, one-dimensional (1D) microrods^8,9^, nanorods^10–14^, and nanowires^15^. The BiSI crystals consist of the [(BiSI)_∞_]_2_ double chains bonded together by the weak van der Waals interactions^1,16^. The chains are oriented along the *c*-axis, i.e. [001] direction^5,13,17^. Therefore, this material possesses highly anisotropic optical and electrical properties. The BiSI is n-type semiconductor^8,9,18,19^ with energy band gap reported in the wide range from 1.5 eV^20^ up to 1.8 eV^15,21,22^. BiSI is considered as an efficient solar absorber for photovoltaic devices^8,21^. It has been demonstrated as an excellent photoconductor with large photoconductive gain^17,23^. Moreover, it exhibits small effective mass of electrons and holes which is beneficial for its use in room temperature radiation detectors^1,24^. The BiSI is also a ferroelectric material^25–27^. Recently, an intrinsic ultra-low lattice thermal conductivity of orthorhombic BiSI has been revealed suggesting that this compound is promising for thermoelectric applications^28^. Until now, the BiSI has been reported as an excellent material for use in high performance photodetectors^17^, solar cells^2,9,11,13,20,29,30^, photoelectrochemical cells^8,19,31^, supercapacitors^3,4,32,33^, rechargeable batteries^16^, room temperature ionizing radiation detectors^10,12^, photocatalytic degradation of organic pollutants^15,34,35^, and hydrogen production^36^.

The BiSI can be fabricated using different approaches, including solid-state mechanochemical method^37,38^, hydrothermal growth^22,34,35,39^, solvothermal synthesis^10,12,14,18^, solution precipitation method^32^, colloidal approach^19^, thermolysis^20^, vapor phase growth^5,6^, and sulfurization of the bismuth oxyiodide (BiOI) in presence of diluted H_2_S gas via anion exchange of the oxygen with sulfur^17,31^. Usually, a synthesis of BiSI nanorods is accompanied with formation of minor phase of rod-like Bi_13_S_18_I_2_^18^ or sheet-like BiOI^32^, depending on applied fabrication method. Li and coworkers^18^ demonstrated that the BiSI can be synthesized under low sulfur to bismuth ratio. When this parameter is increased significantly, the BiSI is converted into the Bi_13_S_18_I_2_. Many of the aforementioned fabrication methods result in formation of textured thin films with random crystal orientation^40^. The one-dimensional BiSI microstructures grow in the natural environment, too. The BiSI is known also as demicheleite-(I) mineral. In 2010, it was discovered in La Fossa crater on the Vulcano Island (Italy)^41^.

Recently, Zankat et al.^42^ have developed self-powered photodetector based on SnSe_2_/MoSe_2_ heterostructure. An influence of MoSe_2_ crystal anisotropy on self-powered photodetection of SnSe_2_/MoSe_2_ heterojunction was investigated. The device exhibited type-II junction with high photoresponsivity of 7.09 A/W, detectivity of 6.44 × 10^12^ Jones, and ON/OFF ratio of 10^5^–10^6^^42^. Patel et al.^43^ illustrated the ability to use p-WSe_2_/p-CuO heterostructure to make a flexible, robust, and broadband photodetector at a low cost. The WSe_2_/CuO thin film was deposited on the paper substrate using a non-toxic, solvent-free, and environmentally friendly handprint process. This paper-based photodetector showed an effective optoelectrical performance over extended spectral range of 390–800 nm with a considerable responsivity of 0.28 mA/W and specific detectivity of 0.19 × 10^10^ Jones^43^. The sonication assisted mechanical mixing and drop-casting technique were presented in^44^ and used to construct a hybrid junction of selenium and poly (3,4-ethylenedioxythiophene) polystyrene sulfonate (PEDOT:PSS). This heterojunction was applied as a high-performance photodetector. It demonstrated a broad spectrum response in the UV–Vis-NIR region with responsivity of 0.56 A/W, 66 mA/W, and 1.363 A/W at wavelength of 315 nm, 620 nm, and 820 nm, respectively^44^. Chekke et al.^45^ fabricated self-powered flexible and wearable single-electrode triboelectric nanogenerator device using Au nanoparticle decorated WS_2_ nanosheets, cellulose paper, and polyvinyl alcohol (PVA) membrane substrate. It exhibited a photo-detection property with a sensitivity of 0.4 Vm^2^/W. Vuong et al.^46^ showed that chemical-vapor-deposited methylammonium bismuth iodide [MA_3_Bi_2_I_9_ (MBI)] films and their mixed halide analogues [MA_3_Bi_2_I_6_Br_3_ (MBIB), MA_3_Bi_2_I_6_Cl_3_ (MBIC)] improve the performance and stability of photodetectors. When MBIC-integrated devices were illuminated with UV light, they showed responsivity of 0.92 A/W and detectivity of 2.9 × 10^13^ Jones, which were approximately three times greater than MBI counterparts^46^. Patel and co-workers demonstrated the fabrication of a flexible film of Ag nanoparticle decorated WSe_2_ on a paper substrate^47^. This material was utilized in a photodetector which responsivity and detectivity at a low bias of 1 V attained 0.43 mA/W and 2.9 × 10^8^ Jones, respectively^47^. Pataniya et al.^48^ developed a dip-coated WSe_2_ photodetector on Whatman filter paper as the substrate. Its responsivity reached 17.78 mA/W under 5 V bias voltage, which was equivalent to previous two-dimensional transition metal dichalcogenides photodetectors on rigid substrates. In another work, Modi et al.^49^ employed straightforward hydrothermal method to synthesize indium-doped SnS ternary alloys. The best photodetector performance was achieved for 7% In doped SnS. The large responsivity of 85 A/W and detectivity of 8.96 × 10^10^ Jones were determined for this photodetector at 1 V bias voltage under illumination intensity of 6.96 mW/m^2^^49^.

In this paper, a facile wet chemical fabrication method of BiSI nanorods is presented. It allowed to obtain high purity material at relatively low temperature (393 K) without a need of application of complex and expensive equipment. The comprehensive studies of morphology, chemical composition, crystal structure, and optical properties of the BiSI nanorods were performed using different experimental techniques, such as high resolution transmission microscopy (HRTEM), scanning electron microscopy (SEM), energy-dispersive X-ray spectroscopy (EDS), X-ray diffraction (XRD), and diffuse reflectance spectroscopy (DRS). The BiSI nanorods were used to construct two types of photodetectors. The first one was traditional photoconductive device which consisted of the BiSI film on stiff glass substrate. The second one was the flexible photo-chargeable photodetector based on the BiSI and gel electrolyte films clamped in between ITO coated PET layers. The response of the photodetectors to monochromatic light (λ = 488 nm, 632.8 nm) illumination was measured. An influence of light intensity on photocurrent response was investigated. The parameters describing photodetectors performance were determined and discussed.

## Methods

### Material synthesis

**Figure 1:**
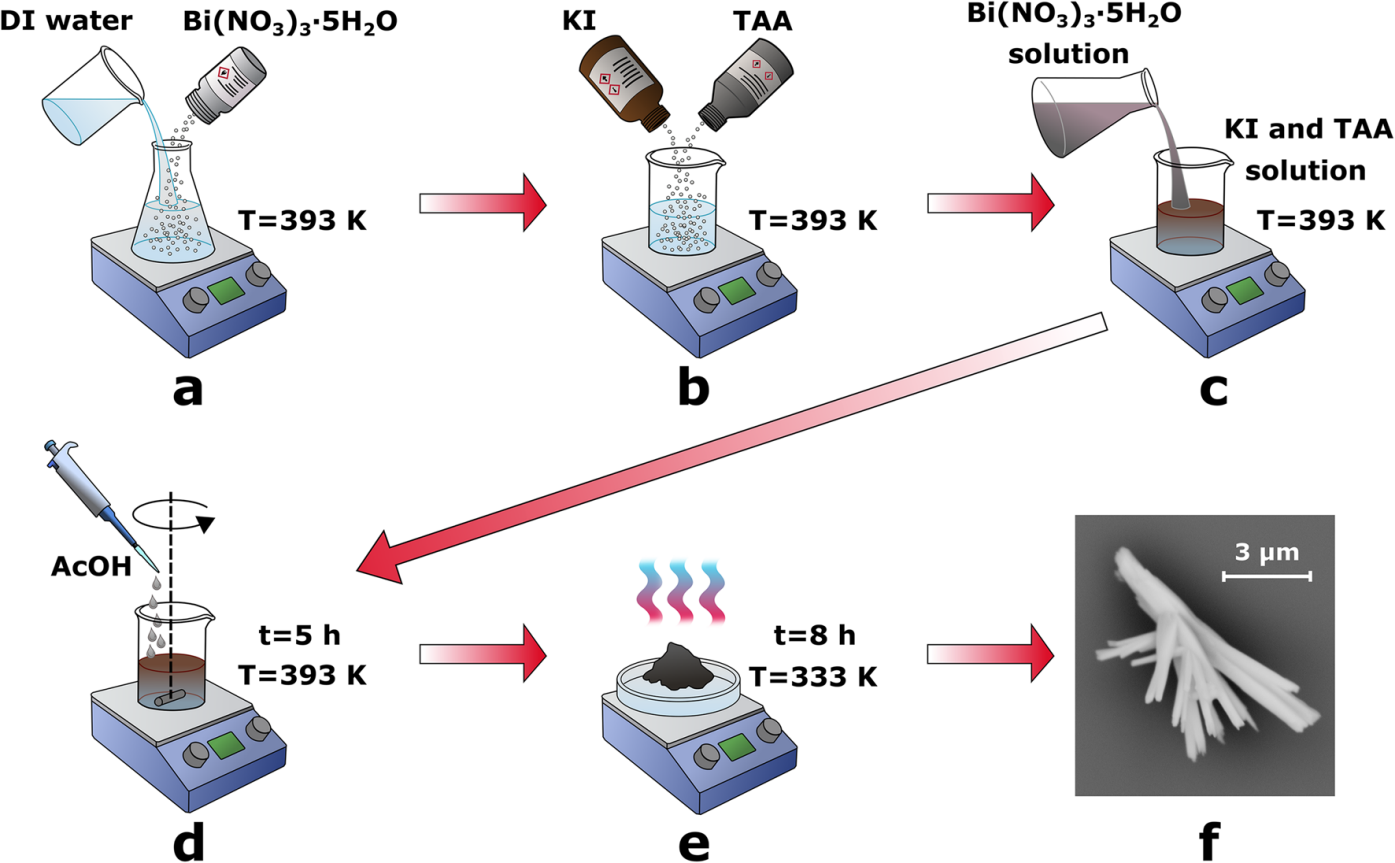
Schematic diagram of the material synthesis: (**a**) bismuth nitrate dissolved in deionized water, (**b**) potassium iodide and thioacetamide dissolved in deionized water, (**c**) the bismuth nitrate solution added to potassium iodide and thioacetamide mixture, (**d**) the solution stirring along with pH adjusting by adding the acetic acid, (**e**) material drying, and (**f**) SEM micrograph of the prepared material. Detailed description is provided in the text.

A typical process of the material fabrication is depicted in Fig. 1. In the first step, 0.485 g of bismuth nitrate pentahydrate (Bi(NO_3_)_3_·5H_2_O) was dissolved in 50 mL of deionized (DI) water and heated to 393 K (Fig. 1a). Then, 0.34 g of potassium iodide (KI) and 1.0 g of thioacetamide (TAA) were dissolved in 50 mL of DI water and heated to 393 K (Fig. 1b). The Bi(NO_3_)_3_·5H_2_O solution was slowly added to the mixture of KI and TAA (Fig. 1c). The pH value of the solution was adjusted to 1–1.2 by adding an appropriate amount of acetic acid (AcOH). The reaction was continued for next 5 h at 393 K under continuous stirring condition (Fig. 1d). After completion of the reaction, the precipitate was washed and centrifuged several times with ethanol (4 times) and deionized water (6 times) until the supernatant liquid became colorless. Later, the precipitate was dried at 333 K for 8 h (Fig. 1e). Finally, the black powder containing one-dimensional BiSI nanorods was obtained (Fig. 1f).

### Characterization of material morphology, chemical composition, crystal structure, and optical properties

The morphological analysis and elemental mapping of the BiSI nanorods were accomplished using bright field imaging in JEM-2100F TEM microscope (JEOL). The acceleration voltage was adjusted to 200 kV. Further characterization of the morphology and chemical composition of the prepared material was carried out with a Phenom Pro X (Thermo Fisher Scientific) SEM microscope integrated with EDS spectrometer. The SEM microscope was operated at an acceleration voltage of 15 kV. The EDS spectrum was quantified using a ProSuite Element Identification (Thermo Fisher Scientific) software.

XRD studies were performed using the D8 Advance diffractometer (Bruker) with Cu-Kα cathode (λ = 1.54 Å) operating at 40 kV voltage and 40 mA current. The scanning step of 0.02° with a scan rate of 0.40°/min in the angle (2Θ) range from 10° to 120° was used. The DIFFRAC.EVA program and International Centre for Diffraction Data (ICDD) PDF#2 database were applied to identify the phases in the XRD spectrum. The exact lattice parameters and crystallite size of fitted phases were calculated using Rietveld refinement in TOPAS 6 program, basing on Williamson-Hall theory^50,51^. The pseudo-Voigt function was applied for a description of diffraction line profiles at the Rietveld refinement. The weighted-pattern factor (*R*_*wp*_), expected *R* factor (*R*_*exp*_) and goodness-of-fit (*GOF*) parameters were used as numerical criteria of the quality of the fit of calculated to experimental diffraction data^52^. Peak shapes, lattice parameters, crystallite size and lattice strain were refined simultaneously^50,51,53^.

DRS spectrum of the BiSI nanorods was recorded at room temperature using PC-2000 spectrophotometer (Ocean Optics Inc.) connected to the ISP-REF integrating sphere (Ocean Optics Inc.). The sample for optical measurements was prepared as follows. A small amount of material was added to ethanol and agitated ultrasonically for 30 min. Then, the suspension of BiSI nanorods in ethanol was drop casted on a glass substrate multiple times. The material deposition was continued until the glass substrate was fully coated with BiSI. After that, the sample was dried at room temperature to evaporate the ethanol.

### Preparation and examination of the BiSI based photodetectors

Two types of photodetectors were constructed. The first one was fabricated as follows. The BiSI nanorods were dispersed in ethanol and agitated ultrasonically for 1 h. Afterwards, the BiSI suspension in ethanol was drop casted onto the glass plate and dried. This process was repeated multiple times until the glass plate was fully coated with BiSI. The gold electrodes with a distance of 385 µm were sputtered on the BiSI film using Q150R ES rotary pumped coater (Quorum Technologies Ltd.). The gold layers were chosen as the materials for the photodetector electrodes due to their high quality and chemical stability^54^. Thin metal wires were attached to the sample electrodes with a high purity silver paste. The second type of photodetectors was prepared according to the procedure described below. The BiSI nanorods (200 mg) were dispersed in ethanol (12 mL) and agitated ultrasonically. The suspension of BiSI nanorods in ethanol was drop casted onto polyethylene terephthalate (PET) substrate coated with indium tin oxide (ITO) layer. Then, the sample was dried. The drop casting was repeated for 20 times to achieve a dense BiSI layer on the ITO electrode. In the next step, the sample was heated at temperature of 333 K for 1 h in order to evaporate the residual ethanol. The potassium hydroxide (KOH) (1 g) was dissolved in deionized water (6 mL) and stirred for 1 h at 333 K. The poly(vinyl alcohol) (PVA) (1.5 g) was dissolved in deionized water (10 mL) and stirred for 1 h at 353 K. The aqueous solutions of KOH and PVA were mixed together and heated at temperature of 353 K. A piece of filter paper (AeroPress) with average pore size of 20 µm was placed on the PET/ITO/BiSI sample. It served as a separator which was infiltrated with PVA-KOH solution. The ITO coated PET was attached to the top of the sample. In order to ensure a good connection between BiSI, PVA-KOH, and ITO layers, the sample was clamped into small clips. In order to obtain solidified gel electrolyte, the PET/ITO/BiSI/PVA-KOH/ITO/PET sample was subjected to elevated temperatures of 353 K and 323 K for 1.5 h and 12 h, respectively.

The fabricated samples were inserted into the H-242 environmental test chamber (Espec) and tested as photodetectors. The measurements of photoelectric properties of BiSI nanorods were accomplished at a constant temperature of 293 K and relative humidity (*RH*) of 50%. The photoelectric response of the BiSI nanorods was registered at a constant bias voltage using Keithley 6517B electrometer (Tektronix). In the case of the Au/BiSI/Au photodetector, the bias voltage of 50 V was applied. Such value of bias voltage (or even higher) was commonly used for other photodetectors^55–58^. Furthermore, an application of higher voltage results in achieving of larger photocurrent response of photodetector. It allows also to reduce noise and increase the precision of measurements. The data acquisition was carried out using a PC computer and LabView program (National Instruments). The BiSI based photodetector was illuminated with blue (λ = 488 nm) and red (λ = 632.8 nm) light emitted by argon laser Reliant 50 s (Laser Physics) and helium neon laser 25-LHP (Melles Griot), respectively. The radiation was transmitted from laser to the photodetector using the UV–VIS optical fiber. The neutral filters were applied to adjust the light intensity.

## Results and discussion

### TEM investigations

**Figure 2:**
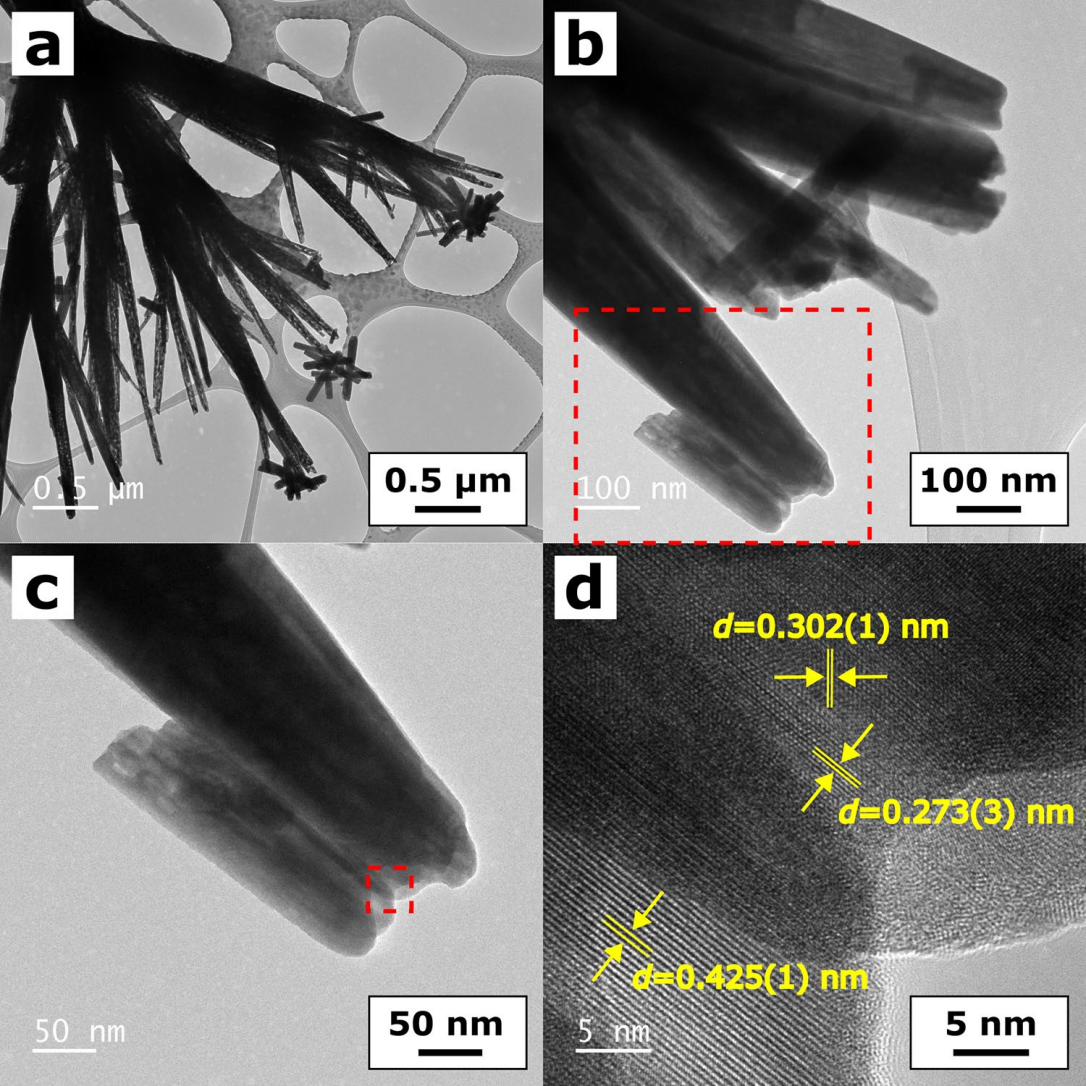
TEM images of the BiSI nanorods (**a-d**) recorded at different magnifications. The figures (**c,d**) represent the magnified areas marked by the red dashed rectangles in figures (**b,c**), respectively. The lattice fringes of 0.425(1) nm, 0.302(1) nm, and 0.273(3) nm correspond to the interplanar distances between (200), (121), and (310) planes of the orthorhombic BiSI (diffraction card No. PDF 00-043-0652).

Figure 2 presents TEM images of the prepared material. The BiSI exhibited one-dimensional structure with lengths from a few hundreds of nanometers up to a several micrometers (Fig. 2a). The clear lattice fringes were observed in the HRTEM image of the nanorods tips (Fig. 2d). Determined interplanar distance *d* = 0.425(1) nm was equal within an experimental uncertainty to the distance of 0.4259 nm between (200) planes in the orthorhombic BiSI (PDF 00-043-0652). The same interplanar distance was observed in the HRTEM images of the BiSI nanorods prepared via solvothermal method^13,18^. The lattice fringes of 0.302(1) nm and 0.273(3) were identified as interplanar distances of 0.3027 nm and 0.2736 nm between (121) and (310) planes, respectively. It allowed to confirm that the nanorods, shown in Fig. 2, belong to the pure orthorhombic BiSI. The lattice fringes corresponding to the (121) crystallographic plane of BiSI were also reported in the case of BiSI nanorods fabricated from solution^4,14^ and through solvothermal method^12^. The elemental mapping of the nanorods bundle is presented in Fig. S1 in the “Supplementary data”. The expected elements (bismuth, sulfur, and iodine) were uniformly distributed in the BiSI nanorods. It suggested the formation of the pure BiSI phase.

### SEM and EDS studies

**Figure 3:**
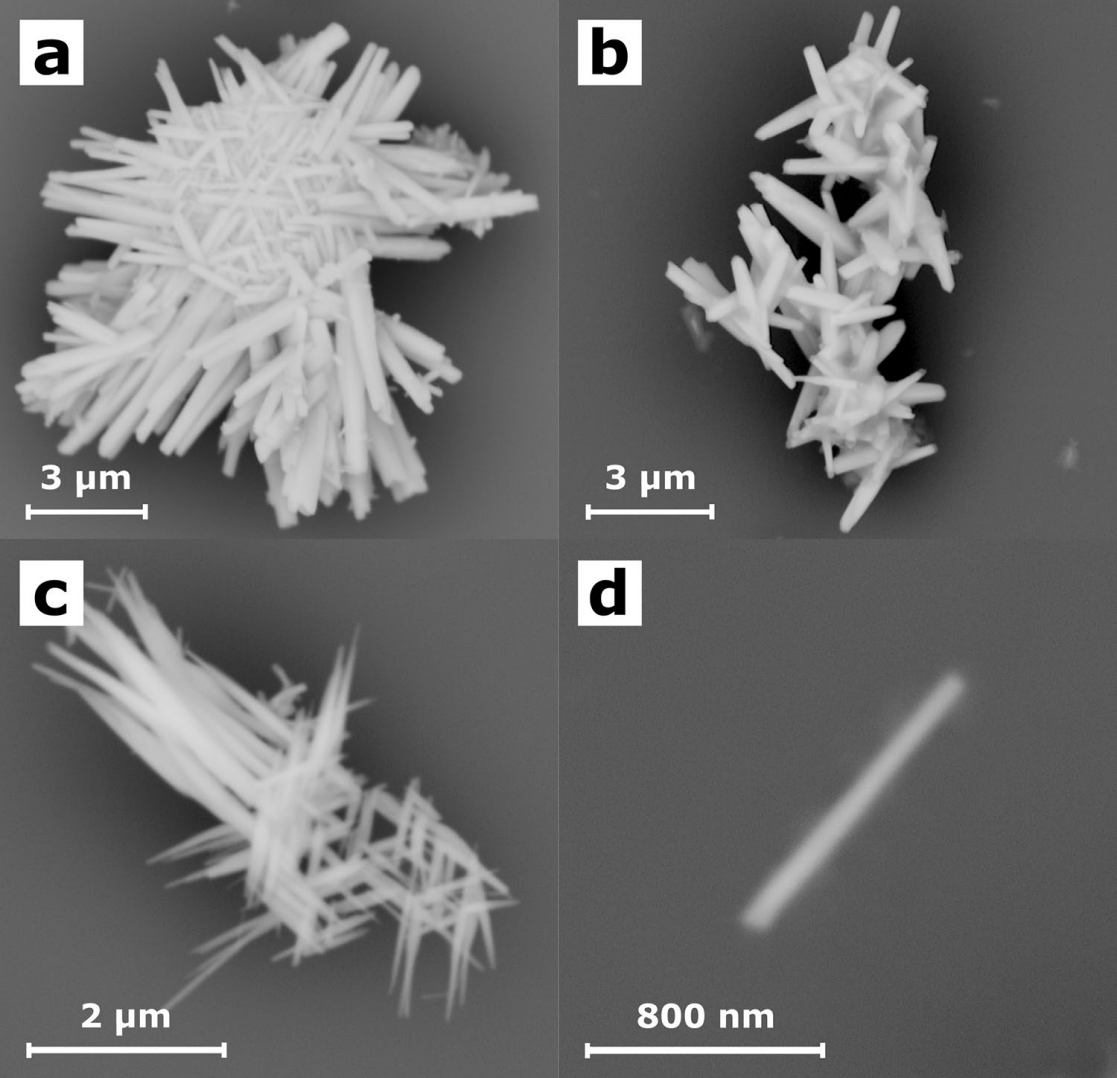
Typical SEM micrographs of the BiSI nanorods bundles (**a–c**) and an individual BiSI nanorod (**d**) deposited on Si substrate.

The prepared material was deposited on the silicon wafer and examined using SEM microscopy (Fig. 3). The material consisted of the crystalline rod-like or needle-like nanostructures with a random arrangement. The BiSI nanorods had tendency to be agglomerated into the bundles (Figs. 3a–c). However, the separate nanorods were observed, too. A typical individual BiSI nanorod with diameter of 73 nm and length of 1.09 µm is depicted in Fig. 3d. The observed growth of the material into bundled one-dimensional nanorods is in agreement with the BiSI crystal structure as reported in the literature. The BiSI possesses the form of a binary screw axis linked together by a strong Bi-S covalent bond, whereas the halogen anion has an ionic bond with a covalent binding bridge^1^. The [(BiSI)_∞_]_2_ double chains are connected by the weak van der Waals interactions and they are oriented along the c-axis^13^.

**Figure 4:**
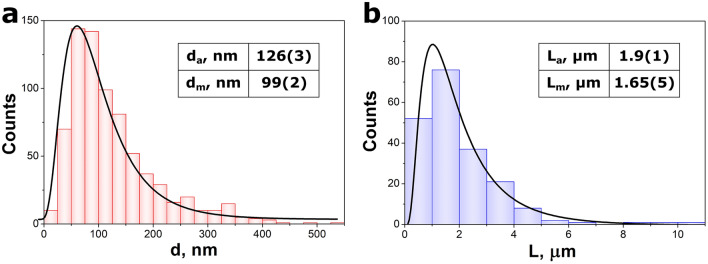
Distribution of diameters (**a**) and lengths (**b**) of the BiSI nanorods. The black lines represent log-normal distribution as described by Eq. (1). The fitted parameters of Eq. (1) are provided in the text. The inset tables show determined average and median values of BiSI nanorods diameters and lengths.

SEM and TEM images were analyzed in order to determine distribution, average values, and median values of the BiSI nanorods dimensions. The measurements of diameters and lengths were performed on 750 and 250 randomly selected nanorods, respectively. It was found that the distribution of the BiSI dimensions (Fig. 4) followed well a log–normal function^59,60^

1 $$f\left(x\right)=\frac{A}{\sqrt{2\pi \cdot }\sigma \cdot x}\mathrm{exp}\left[-\frac{{\left[\mathrm{ln}\left(\frac{x}{{x}_{m}}\right)\right]}^{2}}{2{\sigma }^{2}}\right],$$

where *x* denotes the nanorod dimension (diameter or length), *x*_*m*_ is the median value of the nanorod dimension, σ means a standard deviation, *A* is a constant parameter. Usually, the log–normal function describes sizes distribution of nanorods^14,61–64^, nanowires^65,66^, as well as nanoparticles^60,67,68^. The diameters of the BiSI nanorods were observed in a broad range from about 15 nm up to 530 nm, whereas the majority of them varied between 50 and 100 nm (Fig. 4a). The average and median values of nanorods diameters were equal to *d*_*a*_ = 126(3) nm and *d*_*m*_ = 99(2) nm, respectively. The lengths of BiSI nanorods were in the range from approximately 190 nm to 10.2 µm (Fig. 4b). The most of nanorods were longer than 1 µm and shorter than 2 µm. The average length of *L*_*a*_ = 1.9(1) µm and median length of *L*_*m*_ = 1.65(5) µm were determined.

Table 1 shows an overview of the sizes of BiSI one-dimensional nanostructures reported in the literature. The BiSI nanorods, presented in this paper, exhibited the diameter range similar to those prepared using solvothermal method^10,12–14,36^. However, the BiSI nanorods, described herein, were statistically shorter than other 1D BiSI nanostructures^3,4,18,39^. This difference might result from the various synthesis conditions. Both temperature^69^ and time^70,71^ of synthesis can influence the length of the nanorods. It should be underlined that hydrothermal and solvothermal methods require use of high temperature (typically 453 K^10,12,14,36^) and long reaction time (15–30 h^3,4,10,12,14,15,39^). In our approach, proposed in this work, the synthesis temperature and time are significantly reduced to 393 K and 5 h, respectively. Furthermore, this fabrication method is a facile and it does not involve use of complex or expensive equipment.

**Table 1:** The diameters (*d*) and lengths (*L*) of one-dimensional nanostructures of BiSI prepared using different methods (*T*_*S*_*—*synthesis temperature; *t*_*S*_*—*time of synthesis).

Material morphology	Method of material synthesis	*T*_*S*_, K	*t*_*S*_, h	*d*, nm	*L*, µm	References
Nanoneedles	Solvothermal synthesis	433	15		20–30	^3^
Needles	Hydrothermal synthesis	433	15		10–30	^4^
Needles	Wet chemical method	393	1		1–2	^4^
Microrods	Spray pyrolysis	498		500–1000		^8^
Nanorods	Solvothermal synthesis	453	20	184^md^		^10^
Nanorods	Two-step solution process	473	0.5	60–100		^11^
Nanorods	Solvothermal synthesis	453	20	100–200 160(60)^avr^		^12^
Nanorods	Solvothermal synthesis	453	5	50−200	< 4.5	^13^
Nanorods	Solution method	453	1–20	330(40)^avr^		^14^
Nanorods	Solvothermal synthesis	453	1–20	200^md^		^14^
Nanowires	Hydrothermal synthesis	433	30	40–50	0.5	^15^
1D structures	Solvothermal synthesis	468	12	500	20	^18^
nanorods	Solvothermal synthesis	453	10	100−200		^36^
1D structures	Hydrothermal synthesis	473	24	400–2600	10–120	^39^
Nanorods	Wet chemical method	393	5	15–530 126(3)^avr^ 99(2)^md^	0.19–10.2 1.9(1)^avr^ 1.65(5)^md^	This work

The superscripts “avr” and “md” refer to average and median values of nanorods sizes, respectively.

The EDS analysis confirmed that the material consisted of only bismuth (Bi), sulfur (S), and iodine (I) with an elemental atomic ratio of 0.45:0.21:0.34 for Bi, S and I, respectively. The EDS spectrum was corrected by removing the signal originating from silicone (Si) substrate. No other elements were detected indicting high purity of the material. A similar deficiency of sulfur was demonstrated by the X-ray photoelectron spectroscopy (XPS) of the BiSI thin films prepared from single precursor solution^23^ and sulfurization of the BiOI in diluted H_2_S gas^17^. A sulfur-deficient composition was also reported in the case of one-dimensional BiSI nanostructures which were fabricated using solvothermal method^16^. The EDS elemental mapping and line scan of the BiSI nanorods deposited on Si substrate are presented in the “Supplementary data” in Figs. S2 and S3, respectively. The distributions of bismuth, sulfur, and iodine were almost homogeneous over the sample surface and along the BiSI nanorods.

### XRD analysis

**Figure 5:**
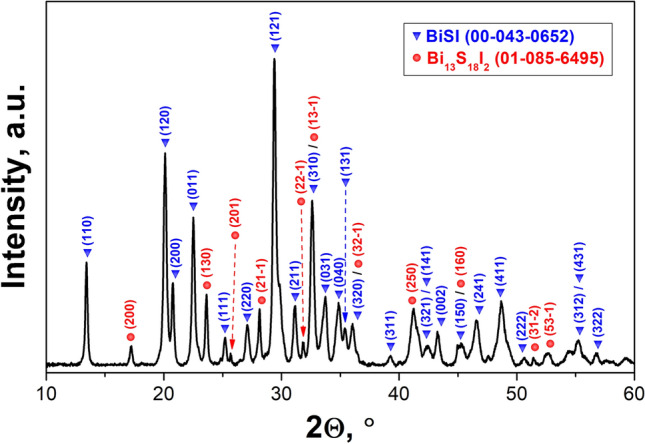
X-ray diffraction pattern of the prepared material. The XRD peaks were identified to BiSI (blue inverted triangle) and Bi_13_S_18_I_2_ (red circle) phases.

X-ray diffraction pattern of the fabricated material is presented in Fig. 5. It consisted of high sharp peaks indicating high crystallinity of the examined material. The orthorhombic BiSI was identified as the main phase. A presence of some residues was also detected. Two strong peaks at 23.8° and 28.1° as well as weak peaks at 17°, 26°, 32°, 45°, 51.6°, 52.5°, and 63° were identified as typical ones for the hexagonal Bi_13_S_18_I_2_^4,72^. Quantitative analysis confirmed a major amount of BiSI phase (87%) and a minor amount of Bi_13_S_18_I_2_ phase (13%), with no presence of other residual phases. The results of Rietveld refinement are provided in Fig. S4 and Table S1 in the “Supplementary data”. A good fit of selected phases to the acquired pattern was obtained. The slight enlargement of the crystal lattice and high lattice strain were observed. These effects can be probably ascribed to the fabrication procedure of the material, resulting in minor misfit of atoms in crystal structure. It should be noted that the growth of the BiSI nanorods from solution is usually accompanied with formation of residual Bi_13_S_18_I_2_^4,18^. Groom and co-workers^72,73^ demonstrated that the iodine concentration in the S/I_2_ flux and temperature of the reaction are crucial parameters that influence the exact amounts of BiSI and Bi_13_S_18_I_2_ in final product.

### DRS measurements

**Figure 6:**
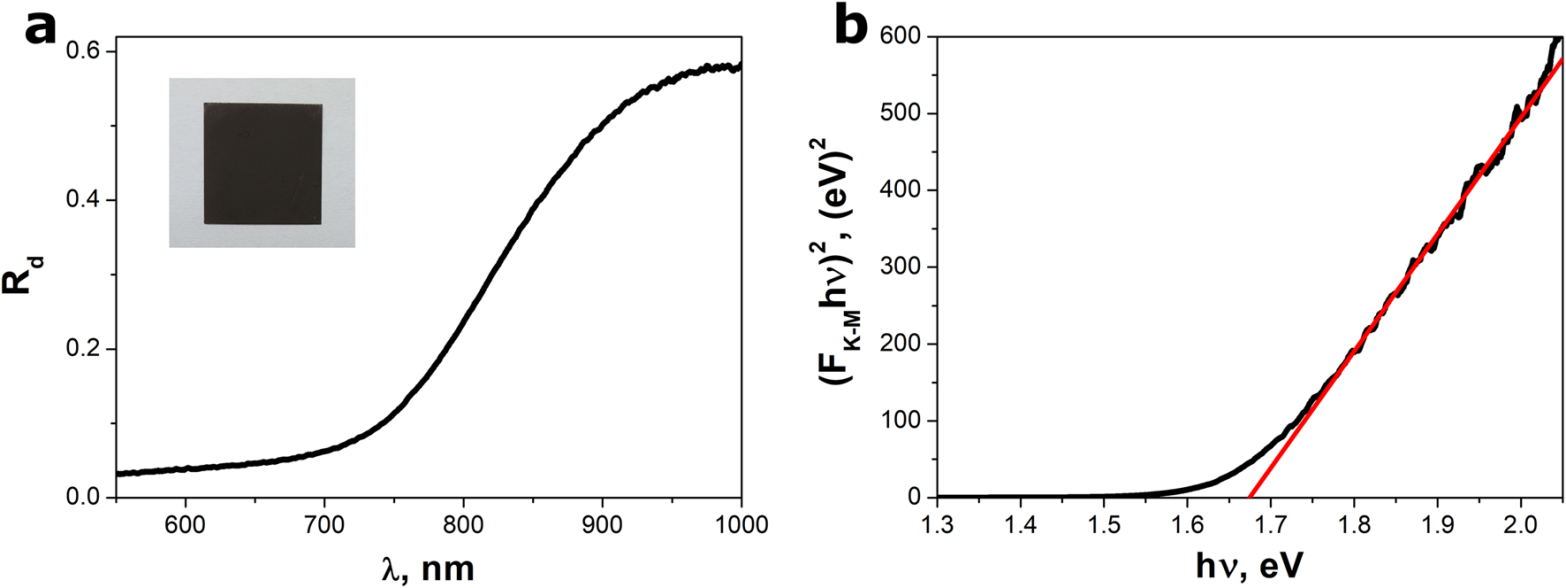
The diffuse reflectance spectrum (**a**) and Tauc plot (**b**) for the BiSI nanorods. An inset in figure (**a**) shows photograph of the BiSI nanorods film deposited on a glass plate. The red curve in figure (**b**) represents the best fitted dependence described by Eq. (3).

Diffuse reflectance spectrum of the BiSI nanorods is presented in Fig. 6a. It showed a clear absorption edge at photon wavelength of about 750 nm. The values of diffuse reflectance coefficient (*R*_*d*_) were converted into the Kubelka–Munk function using well known equation

2 $${F}_{K-M}=\frac{{\left(1-{R}_{d}\right)}^{2}}{2{R}_{d}}.$$

The Kubelka–Munk function is proportional to the absorption coefficient^74^. The band gap energy (*E*_*g*_) of examined material was determined by applying Tauc’s formula^18,32^

3 $${\left({F}_{K-M}\cdot hv\right)}^{1/n}=A\left(hv-{E}_{g}\right),$$

where *hν* is incident photon energy, *A* and *n* are constants. The exponent *n* is equal to 1/2 or 2 in the case of the allowed direct or indirect transitions, respectively. The value of *n* was set to 1/2 since BiSI is regarded as a semiconductor with direct energy band gap^8,17,20,23^. The energy band gap of 1.67(1) eV was determined by extrapolating the straight line to zero absorption in the graph of transformed Kubelka–Munk function versus photon energy (Fig. 6b). The calculated value of *E*_*g*_ was compared with literature data for BiSI (Table 2). One can see that the energy band gap of BiSI is reported in broad range from 1.33 eV^32^ to 1.8 eV^15,22,37^. The value of energy band gap of BiSI may depend on many factors, including material morphology^75^, size of the micro/nanostructures^76,77^, and material thickness^17^. The determined *E*_*g*_ value allows to clearly identify the main phase of examined material as BiSI, since the indirect and direct band gaps of Bi_13_S_18_I_2_ at room temperature are much lower and they equal to 0.73 eV and 1.06 eV^78^, respectively.

**Table 2:** A comparison of the energy band gap (*E*_*g*_) determined for BiSI nanorods with literature data for bismuth sulfoiodide.

BiSI morphology	Type of the band gap	*E*_*g*_, eV	References
Rod-like structures	Allowed indirect	1.33	^32^
Film	Allowed indirect	1.50	^20^
Rod-like particles	Allowed indirect	1.57	^18^
Film	Allowed indirect	1.57	^17^
Film	Allowed indirect	1.57	^8^
Rod-like microstructures	Allowed direct	1.59	^16^
Nanorods	Allowed indirect	1.6	^14^
Film		1.61	^11^
Film	Allowed direct	1.62	^20^
Film	Allowed direct	1.63	^9^
Film	Allowed direct	1.63	^17^
Film	Allowed direct	1.63	^8^
Film	Allowed direct	1.64	^23^
Nanoparticles	Forbidden indirect	1.65	^35^
Nanorods		1.65	^34^
Nanoparticles		1.8	^37^
Microrods		1.8	^22^
Nanowires		1.8	^15^
Nanorods	Allowed direct	1.67(1)	This work

### Examination of the photodetectors

**Figure 7:**
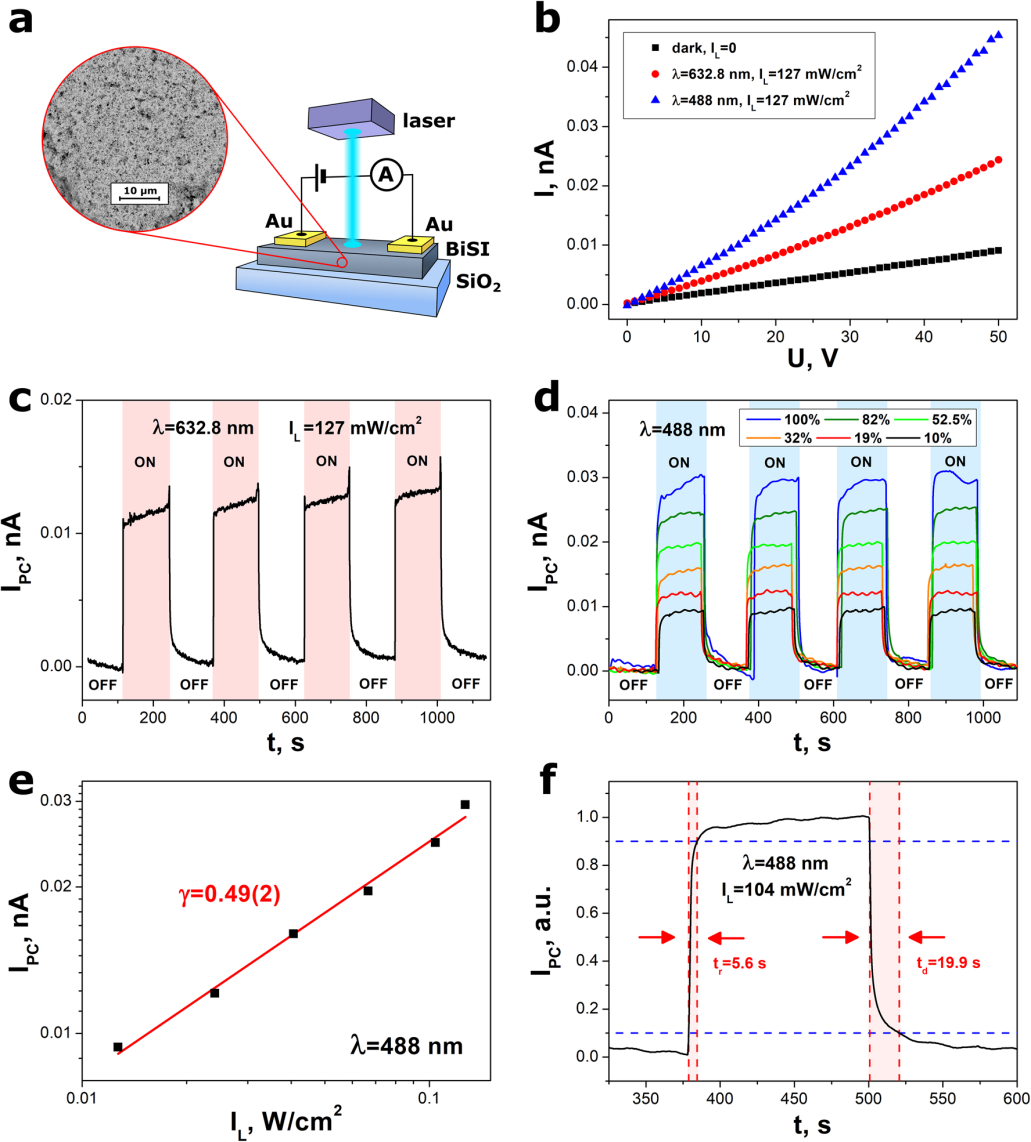
(**a**) A scheme of the biased photodetector consisting of BiSI nanorods film on glass substrate with sputtered Au electrodes, (**b**) current–voltage characteristics of the Au/BiSI/Au photodetector measured in dark condition and under monochromatic light illumination (*I*_*L*_ = 127 mW/cm^2^), (**c**) transient characteristics of photocurrent registered at a constant bias voltage (*U* = 50 V, *T* = 293 K, *RH* = 50%, λ = 632.8 nm, *I*_*L*_ = 127 mW/cm^2^), (**d**) transient characteristics of photocurrent measured for different light intensities at a constant bias voltage (*U* = 50 V, *T* = 293 K, *RH* = 50%, λ = 488 nm, *I*_*Lmax*_ = 127 mW/cm^2^), (**e**) influence of light intensity on photocurrent (λ = 488 nm), (**f**) single cycle of photodetector illumination presenting the rise and fall times (λ = 488 nm, *I*_*L*_ = 104 mW/cm^2^). An inset in figure (**a**) shows SEM image of the BiSI film. The words “ON”, “OFF” in figures (**c,d**) refer to the photodetector illumination and dark condition, respectively. The red line in figure (**e**) represents the best fitted dependence described by Eq. (4).

The two types of photodetectors were investigated. The first one consisted of BiSI film deposited on the glass substrate (Fig. 7a). Figure 7b presents the current–voltage characteristics of this device measured in dark condition and under monochromatic light illumination. The BiSI photodetector was illuminated with blue (λ = 488 nm) and red (λ = 632.8 nm) light to demonstrate its suitability for a full visible spectrum detection. In both cases, the light intensity was the same (127 mW/cm^2^). An existence of the band bending at the Au/BiSI junction is expected. A photocurrent generation in the Au/BiSI/Au device and energy band diagrams in dark condition and under light illumination are presented in Fig. S5 in the “Supplementary data”. The transient characteristics of the photocurrent registered at a constant bias voltage under red (λ = 632.8 nm) and blue (λ = 488 nm) light illumination are shown in Fig. 7c, d, respectively. An influence of light intensity on transient characteristics of the photocurrent was examined (Fig. 7d). An increase of the light intensity resulted in obvious enhancement of the photocurrent. The response of the Au/BiSI/Au photodetector exhibited an excellent repeatability. A stability of the photocurrent response is an important feature of the photodetector^79–83^. It should be underlined that photocurrent response did not show any drift, what proved a good stability of the device operation (Fig. 7d). The dependence of light intensity on photocurrent (Fig. 7e) was best fitted with well-known power law equation^84–87^

4 $${I}_{PC}={I}_{PC0}{\cdot I}_{L}^{\gamma },$$

where *I*_*PC0*_ is a constant, *I*_*L*_ means light intensity, γ is the power exponent that depends on light wavelength. The coefficient γ = 0.49(2) was determined for λ = 488 nm. The value of γ < 1 suggested the photogating effect^88^ as a dominant mechanism of the photocurrent generation. It can be probably ascribed to the existence of the trapping states in the BiSI nanorods^84^.

A typical single cycle of the normalized response of the BiSI photodetector is presented in Fig. 7f. The rise (*t*_*r*_) and fall (*t*_*f*_) times were calculated as the time intervals taken between 10 and 90% of the maximum photocurrent at the rising and recovery edges, respectively^17,84^. Figure S6 in the “Supplementary data” depicts the influence of the light intensity on rise and fall times averaged over multiple ON/OFF cycles of the photodetector illumination (Fig. 7d). An increase of the *I*_*L*_ led to the slight and significant reduction of the *t*_*r*_ and *t*_*f*_, accordingly. This effect was also reported in the case of the other photodetectors based on the BiSI film^17^, Ga_2_O_3_ film^89^, and ZnO nanowires^90^. The rise time *t*_*r*_ = 5.9(16) s and fall time *t*_*r*_ = 14(7) s were determined for the highest light intensity (*I*_*L*_ = 127 mW/cm^2^). It was observed that the rise time is shorter than the decay duration, which strongly suggests that trap and defect states were involved. The Rose's model which proposes that traps and defect states are dispersed with variable concentration in the bandgap, is in good accord with the lowering of the rise and fall time with increasing light intensity. Since the semiconductor is not in a state of thermal equilibrium under illumination, extra electrons and holes are generated in the BiSI nanorods. As a result, two quasi-Fermi levels for electrons and holes are induced. The quasi-Fermi levels for electrons and holes move toward the conduction and valence bands, respectively, as light intensity rises, and an increasing number of traps become recombination sites. In result, the rising and fall times are drastically shortened^91^.

Different figures of merit are used commonly to characterize the sensing performance of the photodetectors, including responsivity (*R*_*λ*_), external quantum efficiency (*EQE*), and detectivity (*D*). These parameters are described by the following equations^92,93^

5 $${R}_{\lambda }=\frac{{I}_{PC}}{{P}_{opt}}=\frac{{I}_{PC}}{{I}_{L}\cdot S},$$

6 $$EQE={R}_{\lambda }\frac{h\cdot c}{\lambda \cdot q},$$

7 $$D=EQE\cdot \frac{\lambda \cdot q}{h\cdot c}\sqrt{\frac{R\cdot S}{4{k}_{B}T}},$$

where *I*_*PC*_ is a photocurrent, *P*_*opt*_ means an optical power density, *I*_*L*_ denotes light intensity, *S* is the effective illumination area of the device, *h* = 6.63 × 10^–34^ J s is Planck’s constant, *c* = 3 × 10^8^ m/s is light velocity, *q* = 1.6 × 10^–19^ C is the elementary charge, *R*·*S* is the resistance area product, *k*_*B*_ = 1.38 × 10^–23^ J/K is Boltzmann constant, and *T* means temperature. The responsivity of 64(2) nA/W, external quantum efficiency of 1.63(5) × 10^–5^%, and detectivity of 1.27(5) × 10^8^ Jones were determined for the Au/BiSI/Au photodetector under blue light illumination (λ = 488 nm, *I*_*L*_ = 12.7 mW/cm^2^). It should be underlined that an increase of light intensity strongly reduces the responsivity, external quantum efficiency, and detectivity of the photodetector^17,84^. Therefore, an application of much smaller light intensity should result in a significant enhancement of *R*_*λ*_, *EQE*, and *D* parameters. Such experiments will be performed in the future.

Table 3 presents the data reported in the literature for photodetectors constructed from various bismuth chalcohalide nanomaterials. The photodetector based on the BiSI nanorods showed shortened rise time than this determined for BiOCl-TiO_2_ heterojunction^87^. Moreover, it exhibited improved γ power coefficient in comparison to the BiSeI micro/nanowires^84^, which proved better sensitivity of the photocurrent response to the change of the light intensity.

**Table 3:** An overview of the photodetectors based on the bismuth chalcohalide nanomaterials prepared using different methods (λ—light wavelength, *I*_*L*_—light intensity, γ—the power exponent, *t*_*r*_—rise time, *t*_*f*_—fall time).

Sensing material	Fabrication method	λ, nm	*I*_*L*_, mW/cm^2^	γ	*t*_*r*_, s	*t*_*f*_, s	References
BiOBr nanosheets	Chemical vapor deposition	365	0.66		0.551	2.416	^99^
BiOI nanosheets	Solution processing	535	0.1		0.99	0.96	^100^
BiOI nanosheets	Chemical vapor deposition	473	100	0.9	0.12	0.25	^85^
Au-BiOI nanosheets	Solution processing	535	0.1		0.97	1.05	^100^
BiOCl nanosheets	Solution processing	350	0.749		1.06	6.87	^86^
ZnO nanoparticle-decorated BiOCl nanosheets	Solution processing	350	0.749	0.782	2.59	0.93	^86^
heterojunction of BiOCl nanosheets and TiO_2_ nanotube	Anodization process and impregnation method	350	2.045	0.607	12.9	0.81	^87^
BiSeI micro/nanowires	Mechanical exfoliation	405		0.26			^84^
		515		0.39			
		488	21.2		0.6	1	
BiSI film	Solution processing	625	70	0.63	0.571	0.112	^17^
BiSI nanorods	Wet chemical method	488	127	0.49(2)	5.9(16)	14(7)	this work

**Figure 8:**
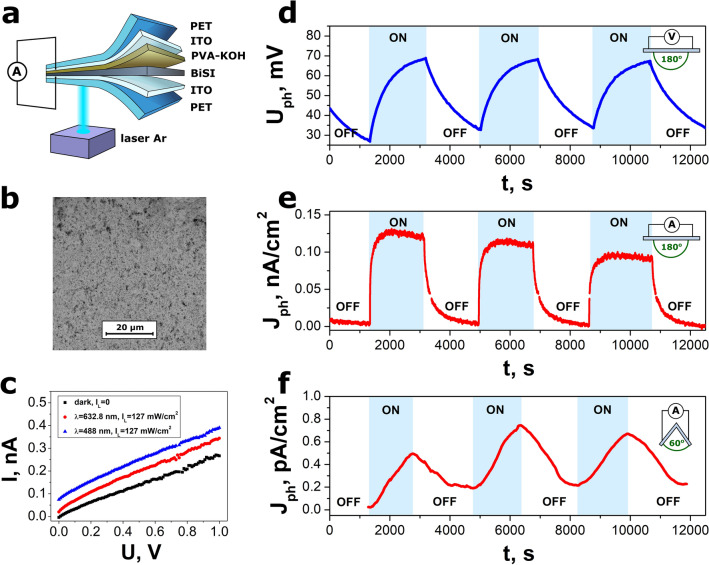
(**a**) A scheme of the flexible photo-chargeable detector consisting of BiSI nanorods film, PVA-KOH gel electrolyte layer and ITO electrodes on PET, (**b**) SEM micrograph of BiSI nanorods film deposited on ITO electrode, (**c**) current–voltage characteristics of the PET/ITO/BiSI/PVA-KOH/ITO/PET photodetector measured in dark condition and under monochromatic light illumination (*I*_*L*_ = 127 mW/cm^2^), transient characteristics of (**d**) open-circuit voltage, short-circuit photocurrent density registered at (**e**) the original state (α = 180°) and (**f**) bent state (α = 60°) of the PET/ITO/BiSI/PVA-KOH/ITO/PET photodetector (*T* = 293 K, *RH* = 50%, λ = 488 nm, *I*_*L*_ = 127 mW/cm^2^).

The second type of examined photodetectors was flexible photo-chargeable BiSI capacitor (Fig. 8a). It consisted of the BiSI nanorods film and PVA-KOH gel electrolyte sandwiched in between the ITO coated PET substrates. The BiSI served as the light absorbing material. The porous structure of the film, composed of randomly oriented BiSI nanorods (Fig. 8b), facilitated higher ion diffusion from the electrolyte^94,95^. Figure 8c presents the current–voltage characteristics of the PET/ITO/BiSI/PVA-KOH/ITO/PET device registered in dark condition and under illumination with blue (λ = 488 nm) and red (λ = 632.8 nm) light. Figure 8d shows transient characteristic of the open-circuit photovoltage of the PET/ITO/BiSI/PVA-KOH/ITO/PET capacitor when no strain was applied to the device (α = 180°). The maximum value of the photovoltage attained 68 mV under monochromatic light illumination (λ = 488 nm, *I*_*L*_ = 127 mW/cm^2^). After the bottom ITO electrode was illuminated (Fig. 8a), the charge carriers were generated inside the BiSI film and participated in the electrolyte ions arrangement^32,96^. The photogenerated electrons were injected into the ITO electrode. Since only one side of the device was illuminated, the nonuniform distribution of the charge carriers in the both electrodes was occurred leading to formation of the open-circuit photovoltage. The short-circuit current was increased and decreased when Ar laser was turned on and off, respectively (Fig. 8e).

The photoelectric response of the PET/ITO/BiSI/PVA-KOH/ITO/PET capacitor was examined for larger number of ON/OFF cycles with shorter time intervals (Fig. S7 in the “Supplementary data”). It proved an remarkable repeatability of the BiSI photodetector response. However, a small decrease of the amplitude of the short-circuit photocurrent was observed with increasing number of the ON/OFF cycle (Fig. 8e and Fig. S7b). This effect could result from degradation of PVA-KOH gel polymer electrolyte^97^. Time dependences of the photovoltage (Fig. 8d, Fig. S7a) and photocurrent (Fig. 8e, Fig. S7b) registered at the original state (α = 180°) were similar to these reported for other photo-chargeable capacitors^32,96^. The not only quantitatively but also qualitatively different transient response of the BiSI photodetector was measured when the device was bent at the angle of α = 60° (Fig. 8f). A strong influence of bending on a photocurrent response indicated a possibility of application of the device as a deformation sensor. The responsivity, external quantum efficiency, and detectivity of the PET/ITO/BiSI/PVA-KOH/ITO/PET capacitor were calculated using Eqs. (5–7). When capacitor was illuminated with blue light (λ = 488 nm, *I*_*L*_ = 127 mW/cm^2^) and no strain was applied to the device, the figures of merit were following: *R*_*λ*_ = 8.7(8) nA/W, *EQE* = 2.2(2) × 10^–6^%, and *D* = 6.3(6) × 10^6^ Jones.

The photoelectric performance of different photo-chargeable capacitors is presented in Table 4. The majority of these devices are stiff. It limits their potential applications. This drawback was eliminated in the flexible PET/ITO/BiSI/PVA-KOH/ITO/PET photodetector. Furthermore, the photovoltage generated in this device was higher than values of this parameter reported for SiO_2_/ITO/PANI/PVA-H_2_SO_4_/PANI-CNT/PET^98^ and SiO_2_/ITO/BiSI/PVA-KOH/BiSI/ITO/SiO_2_^32^ capacitors.

**Table 4:** The figures of merit of various photo-chargeable devices based on the gel electrolytes (λ—light wavelength, *I*_*L*_*—*light intensity, *U*_*ph*_*—*open-circuit photovoltage, *J*_*ph*_*—*short-circuit photocurrent density).

Device structure	Flexible device	Illumination	*U*_*ph*_, mV	*J*_*ph*_, nA/cm^2^	Reference
SiO_2_/ITO/ZnO/ZnCo_2_O_4_/PVA-KOH/ZnCo_2_O_4_/ZnO/ITO/SiO_2_	No	λ = 365 nm, *I*_*L*_ = 3 mW/cm^2^	350		^96^
SiO_2_/ITO/PANI/PVA-H_2_SO_4_/PANI-CNT/PET	No	sunlight (1 sun), *I*_*L*_ = 100 mW/cm^2^	48		^98^
SiO_2_/ITO/SnS_2_-graphene/PVA-KOH/ITO/SiO_2_	No	sunlight, *I*_*L*_ = 100 mW/cm^2^		9	^101^
PET/Bi_2_O_2_Se-graphene/PVA-KOH/CB/ITO	Yes	sunlight, *I*_*L*_ = 120 mW/cm^2^		400	^102^
SiO_2_/ITO/BiSI/PVA-KOH/BiSI/ITO/SiO_2_	No	sunlight (1 sun), *I*_*L*_ = 100 mW/cm^2^	60	100	^32^
PET/ITO/BiSI/PVA-KOH/ITO/PET	Yes	λ = 488 nm, *I*_*L*_ = 127 mW/cm^2^	68	0.11	this work

*CB* carbon black, *CNT* carbon nanotube, *PANI* polyaniline.

## Conclusions

The BiSI nanorods were fabricated via a facile wet chemical method. The high purity material was prepared at relatively low temperature (393 K) using low-cost and simple equipment. Moreover, the synthesis of the material was completed within 5 h. It is a great advantage in comparison to fabrication of BiSI using hydrothermal or solvothermal methods which require high temperature (typically 453 K) and long reaction time (over 15 h). The BiSI nanorods were characterized by applying many different experimental techniques, including HRTEM, SEM, EDS, XRD, and DRS. The orthorhombic BiSI was identified as the main phase of the synthesized material. The one-dimensional morphology of BiSI nanocrystals was revealed. The distribution of the BiSI nanorods dimensions followed well a log–normal function. The average diameter and length of the BiSI nanorods were equal to 126(3) nm and 1.9(1) µm, respectively. The detected chemical elements (bismuth, sulfur, and iodine) were homogeneously distributed in the BiSI nanorods. The direct energy band gap of 1.67(1) eV was determined and confirmed to be in agreement with literature data for BiSI.

The two types of devices were constructed from BiSI nanorods and tested as photodetectors. The first one was composed of BiSI film deposited on the stiff glass substrate and equipped with Au electrodes. The photocurrent response of the Au/BiSI/Au photodetector under monochromatic light illumination (488 nm) was measured at a constant bias voltage. The response of BiSI photodetector exhibited an excellent repeatability and stability. The influence of light intensity on the photocurrent was found to obey well-known power law. The relatively high power coefficient of 0.49(2) indicated a good sensitivity of the photocurrent response to the change of the light intensity. The second type of investigated photodetectors was flexible photo-chargeable capacitor, which contained the BiSI nanorods film and PVA-KOH gel electrolyte sandwiched between the ITO electrodes. The multilayer PET/ITO/BiSI/PVA-KOH/ITO/PET device was used to detect Ar laser radiation without a need to apply to photodetector an external power supply. The photoelectric response of the device was registered at its original state as well as it was bent at 60^0^. When no strain was applied to the PET/ITO/BiSI/PVA-KOH/ITO/PET capacitor, it generated open-circuit photovoltage of 68 mV and short-circuit photocurrent density of 0.11 nA/cm^2^ under illumination with light intensity of 0.127 W/cm^2^. A strong effect of bending on a photocurrent response was observed. It is promising for future applications of the BiSI capacitor as a deformation sensor. The BiSI nanorods were demonstrated to possess a great potential for use in flexible photo-chargeable capacitors and self-powered photodetectors.

## Supplementary Information

Supplementary Information.

## Data Availability

The datasets used and/or analyzed during the current study available from the corresponding author on reasonable request.
