# Supplementary material for: Bismuth sulfoiodide (BiSI) nanorods: synthesis, characterization, and photodetector application
Source: Sci Rep. 2023 May 31;13:8800. doi: 10.1038/s41598-023-35899-7 (PMC10232605; doi:10.1038/s41598-023-35899-7)
Supplement: Supplementary file 1 — Supplementary Information. [file 41598_2023_35899_MOESM1_ESM.docx]

**Supporting Information**

**Bismuth sulfoiodide (BiSI) nanorods:**

**synthesis, characterization, and photodetector application**

Krystian Mistewicz ^a^*, Tushar Kanti Das ^a^, Bartłomiej Nowacki ^b^, Albert Smalcerz ^c^, Hoe Joon Kim ^d^, Sugato Hajra ^d^, Marcin Godzierz ^e,f^, and Olha Masiuchok ^e,f,g^

^a^ Institute of Physics – Center for Science and Education, Silesian University of Technology, Krasińskiego 8, 40-019 Katowice, Poland

^b^ Department of Industrial Informatics, Faculty of Materials Science, Joint Doctorate School, Silesian University of Technology, Krasinskiego 8, 40-019 Katowice, Poland

^c^ Department of Industrial Informatics, Faculty of Materials Science, Silesian University of Technology, Krasinskiego 8, 40-019 Katowice, Poland

^d^ Department of Robotics Engineering, Daegu Gyeongbuk Institute of Science & Technology (DGIST), Daegu 42988, Korea

^e^ Centre of Polymer and Carbon Materials, Polish Academy of Sciences, M. Curie-Skłodowskiej 34, 41-819 Zabrze, Poland

^f^ International Polish-Ukrainian Research Laboratory Formation and Characterization of Advanced Polymers and Polymer Composites (ADPOLCOM)

^g^ E.O. Paton Electric Welding Institute of the National Academy of Sciences of Ukraine, 11 Kazymyr Malevych Str., 03680, Kyiv, Ukraine

*corresponding authors: [krystian.mistewicz@polsl.pl](mailto:krystian.mistewicz@polsl.pl)


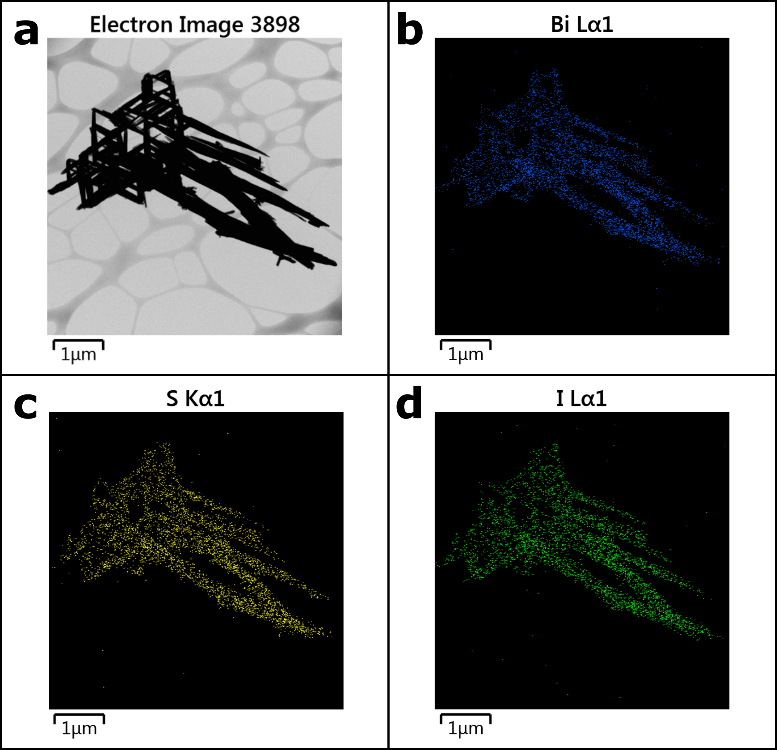


Fig. S1. TEM image (a) of the BiSI nanorods bundle and corresponding elemental mapping for bismuth (b), sulfur (c), and iodine (d).


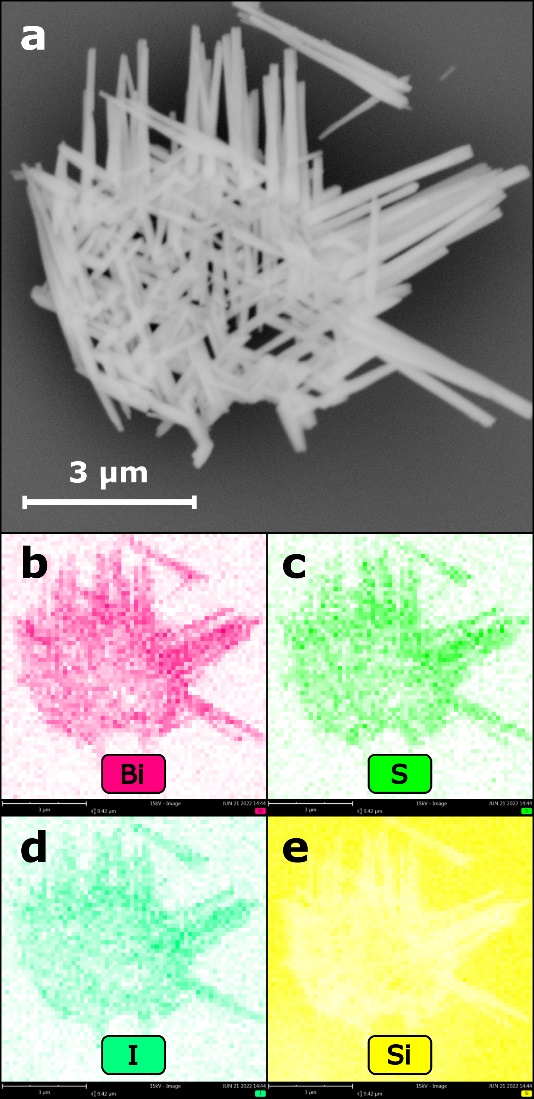


Fig. S2. SEM image (a) of the BiSI nanorods bundle on Si substrate and corresponding elemental mapping for bismuth (b), sulfur (c), iodine (d), and silicon (e).


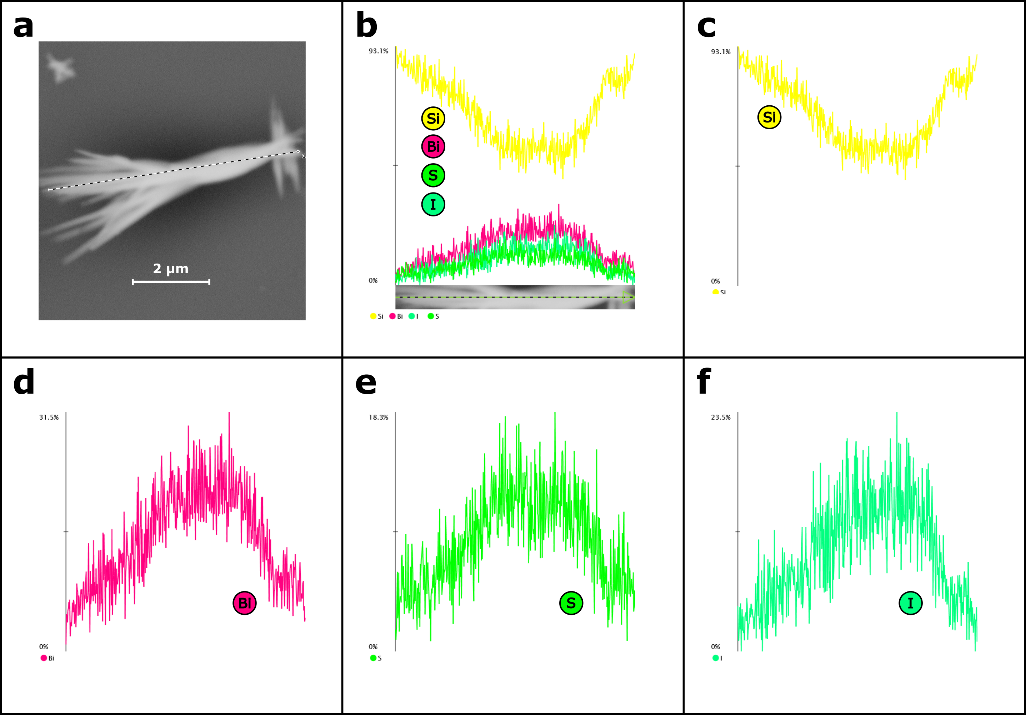


Fig. S3. SEM image (a) of the BiSI nanorods bundle on Si substrate and corresponding EDS line scan of all detected elements (b), silicon (c), bismuth (d), sulfur (e), and iodine (f). The orientation of EDS scan is indicated by dashed line in Figure (a).


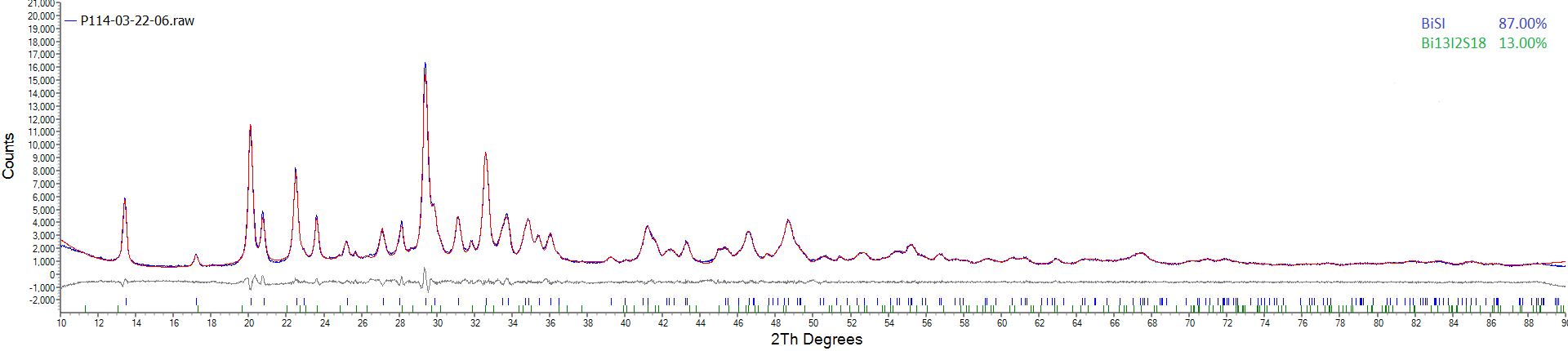


Fig. S4. Results of Rietveld refinement of XRD pattern with marked peaks positions of identified phases. Blue, red, and grey lines represent experimental data, fitting curve, and differential curve, respectively.

Table S1. Structural properties of examined material calculated using Rietveld refinement. The values in brackets represent coefficient of variation, not standard deviation.

| Phase | Space group | Lattice parameters, Å  (ICDD) | Lattice parameters, Å  (calculated) | Crystallite size, nm | Lattice strain,  % |
| --- | --- | --- | --- | --- | --- |
| BiSI  (00-043-0652) | *Pnam* | *a*=8.514,  *b*=10.264,  *c*=4.174 | *a*=8.527,  *b*=10.279,  *c*=4.180 | 53(10) | 0.75(14) |
| Bi_13_S_18_I_2_  (01-085-6495) | *P3* | *a*=15.612,  *c*=4.017 | *a*=15.636,  *c*=4.025 | 57(4) | 0.58(4) |


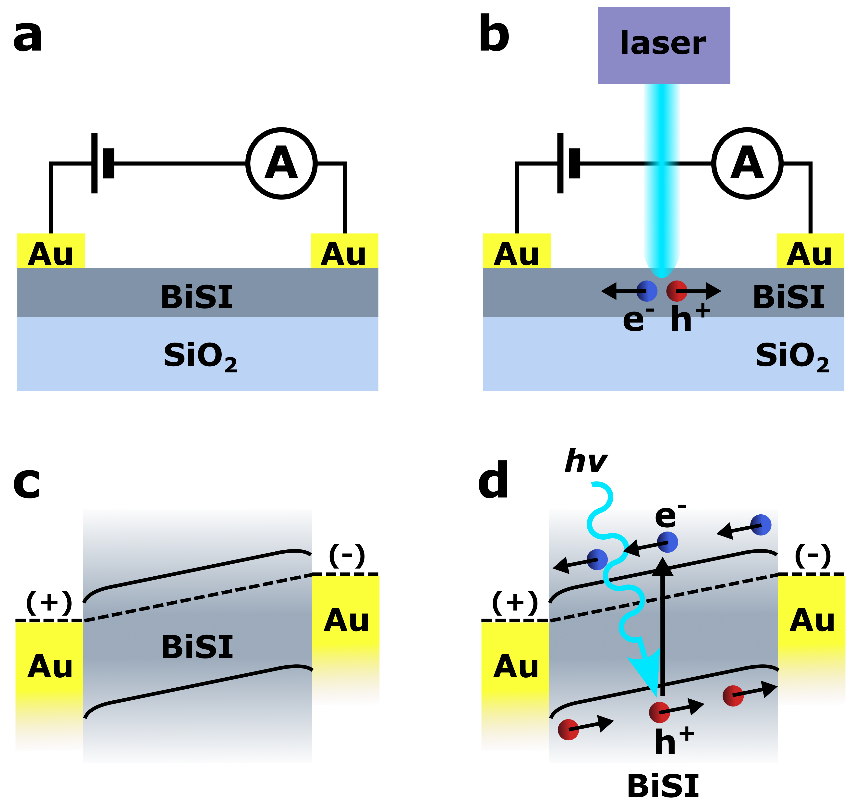


Fig. S5. The schematic illustrations of the Au/BiSI/Au photodetector (a, b) biased at a constant voltage and corresponding energy band diagrams (c, d) in dark condition (a, c) and under light illumination (b, d).





Fig. S6. Influence of light intensity on rise and fall times of the Au/BiSI/Au photodetector (*U*=50 V, *T*=293 K, *RH*=50 %, λ=488 nm).


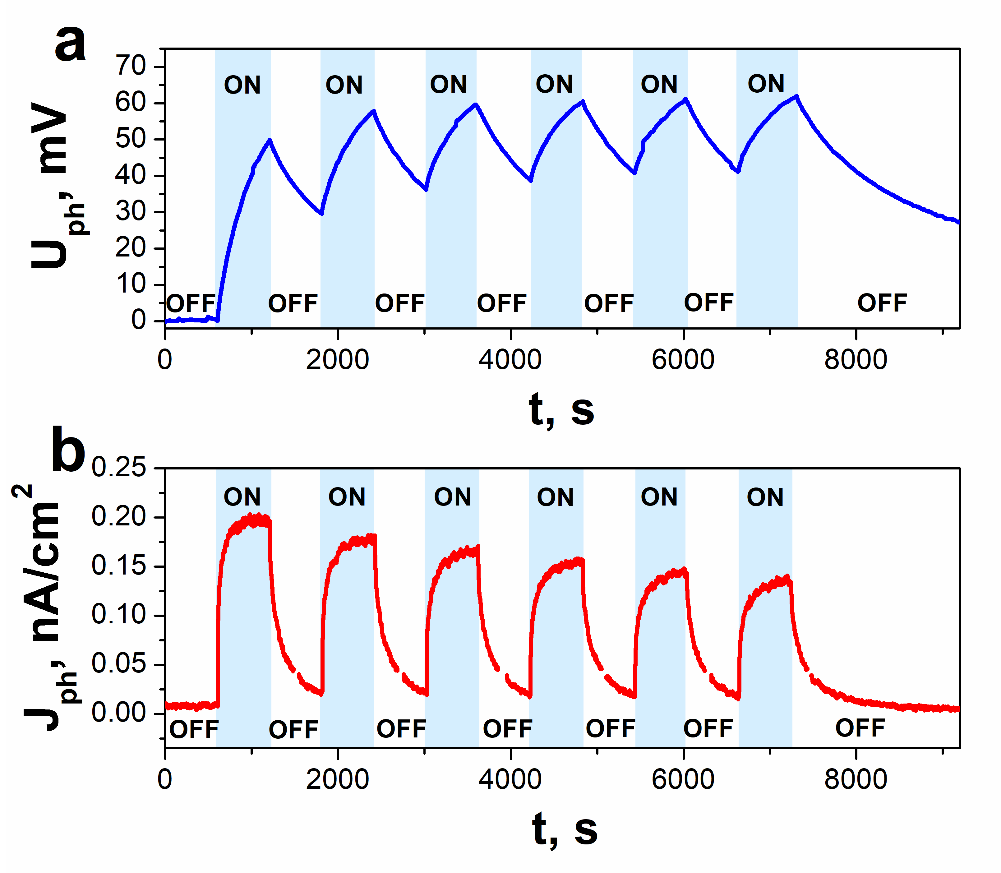


Fig. S7. The open-circuit voltage (a) and short-circuit photocurrent density (b) registered at the original state (α=180^0^) of the PET/ITO/BiSI/PVA-KOH/ITO/PET photodetector (*T*=293 K, *RH*=50 %, λ=488 nm, *I_L_*=127 mW/cm^2^).
